# Biological Motion Processing in Schizotypic and Autistic Traits

**DOI:** 10.1068/ig12

**Published:** 2013-10-01

**Authors:** K S Pilz

**Affiliations:** University of Aberdeen, UK

## Abstract

Previous research has shown that motion processing is significantly impaired in both autistic and schizophrenic patients. One specific area of motion perception that is affected by both disorders is the processing of biological motion. Stimuli that are often used to investigate biological motion perception are point-light walkers (PLW), for which point-lights are attached to the joints of a moving person, which alone are enough to perceive the human figure. PLWs are useful stimuli to investigate biological motion perception, because they contain the local motion information of each dot, and in addition, by grouping the local elements of the walkers into a global form, they have a high-level interpretation of a moving person (normal walker). By scrambling the positions of the points on the screen, the local motion of each dot is preserved while obscuring the underlying form (scrambled walker). Similarly, by randomizing the position of the dots along the underlying skeleton each frame of the moving sequence, the local image motion is lost, whereas the global form of the walker is preserved (random-position walker). Also inverting the walker in a way that it appears to be walking on the ceiling can obscure the global form of the walker (inverted walker). Using these four kinds of walkers, it is possible to investigate the relative contribution of form and motion information to biological motion perception using one single paradigm. We have successfully used these walkers before to investigate biological motion perception in ageing. Here, we tested differences in biological motion processing in healthy young people with different schizotypic (N=38) and autistic traits (N=35). We asked participants to perform a biological motion direction discrimination task for normal, inverted, scrambled and random-position walkers. In addition, they had to complete questionnaires on autistic and schizotypic traits. Using both reaction time and accuracy measures we found differences for processing local motion and global form information between participants with high or low scores on both questionnaires.

